# The complete mitochondrial genome of *Anas penelope* (Anatidae: Anas)

**DOI:** 10.1080/23802359.2020.1768920

**Published:** 2020-05-29

**Authors:** Xiaoping Sun, Chaochao Hu, Shuang Li, Mengfan Zhai, Wei Liu, Yinlong Zhang

**Affiliations:** a Collaborative Innovation Center of Sustainable Forestry in Southern China of Jiangsu Province, Nanjing Forestry University, Nanjing, China; b Jiangsu Environmental Monitoring Center, Nanjing, China; c Nanjing Normal University, Nanjing, China; d Nanjing Institute of Environmental Sciences, Ministry of Ecology and Environment of the People’s Republic of China, Nanjing, China

**Keywords:** *Anas penelope*, mitochondrial DNA, phylogeny

## Abstract

The complete mitochondrial DNA genome of the Eurasian Wigeon, *Anas penelope*, was mapped by the next-generation sequencing and Mega 7.0. The circular mitogenome (16,596 bp in length) contains 13 protein-coding genes, 2 rRNA genes (12S ribosomal RNA and 16S ribosomal RNA), 22 tRNA genes and a control region. The content of four base pairs of the complete mitochondrial DNA is 28.9% of A, 22.3%of T, 32.7%of C and 16.1% of G. To validate the phylogenetic relationship, 25 published complete mitochondrial genomes of Anseriformesalong with the genome of Terek sandpiper were used to construct the phylogenetic tree.

The Eurasian Wigeon (*Anas penelope*) is a widespread duckspecies estimated to number 2,800,000–3,300,000 individuals (IUCN 2016). The overall population trend of *A. penelope* is decreasing due to recreational impacts, dams, water management, and prion-induced diseases (Lei et al. 2008). Except for some behavioral and habitat research, *A. penelope* is less well studied in literature (Guillemain et al. 2002; Mayhew and Houston 2008). Mitochondrial DNA (mtDNA) is regarded as a useful tool in population conservation, phylogeographic, and phylogenetic studies. The mitochondrial DNA control region of *A. penelope* was examined to assess the genetic differentiation (Kulikova & Zhuravlev 2010). Hence, it is necessary to obtain the complete mitochondrial DNA by the next-generation sequencing. The muscle specimen of *A. penelope* was collected from the coast of Rudong Country, Jiangsu Province, China (32°32′43.42ʺ N, 121°06′09.02ʺ E). A voucher specimen was stored in Nanjing Normal University (NJNU: ANPE20191005), Nanjing, China. The complete genome sequence was aligned by Mega 7.0 and deposited in GenBank (Accession Number: MT304825).

The complete mitochondrial genome of *A. penelope* is circular molecular and 16,596 bp in length. The genome contains 37 genes, including 13 protein-coding genes, 2 ribosomal RNAs, 22 tRNA genes, and a control region (D-loop). Most of the genes were encoded on the H-strand, while ND6 and 8tRNA were encoded on the L-strand. For the 13 PCGs, the most common start codon is ATG, followed byGTG. The termination codon (TAA) is most common and two protein-coding genes (COIII and ND4) use single T as their stop codons, which were presumably completed as TAA by post transcriptional polyadenylation  . The base composition of mtDNA is A(28.9%), G(16.1%), C(32.7%) and T(22.3%), and thus the percentage of G and C (48.8%) was slightly lower than A and T (51.2%).

To confirm the phylogenetic position of *A. penelope* among Anseriformes species, a Bayesian analysis was conducted on the complete mitogenome. It is shown that the phylogenetic relationship of *A. penelope* is very close to the *A. falcate*in the family Anatidae (Figure 1). We hope this study will provide more information for the phylogenetic analyses of Anseriformes in future research.

**Figure 1.**
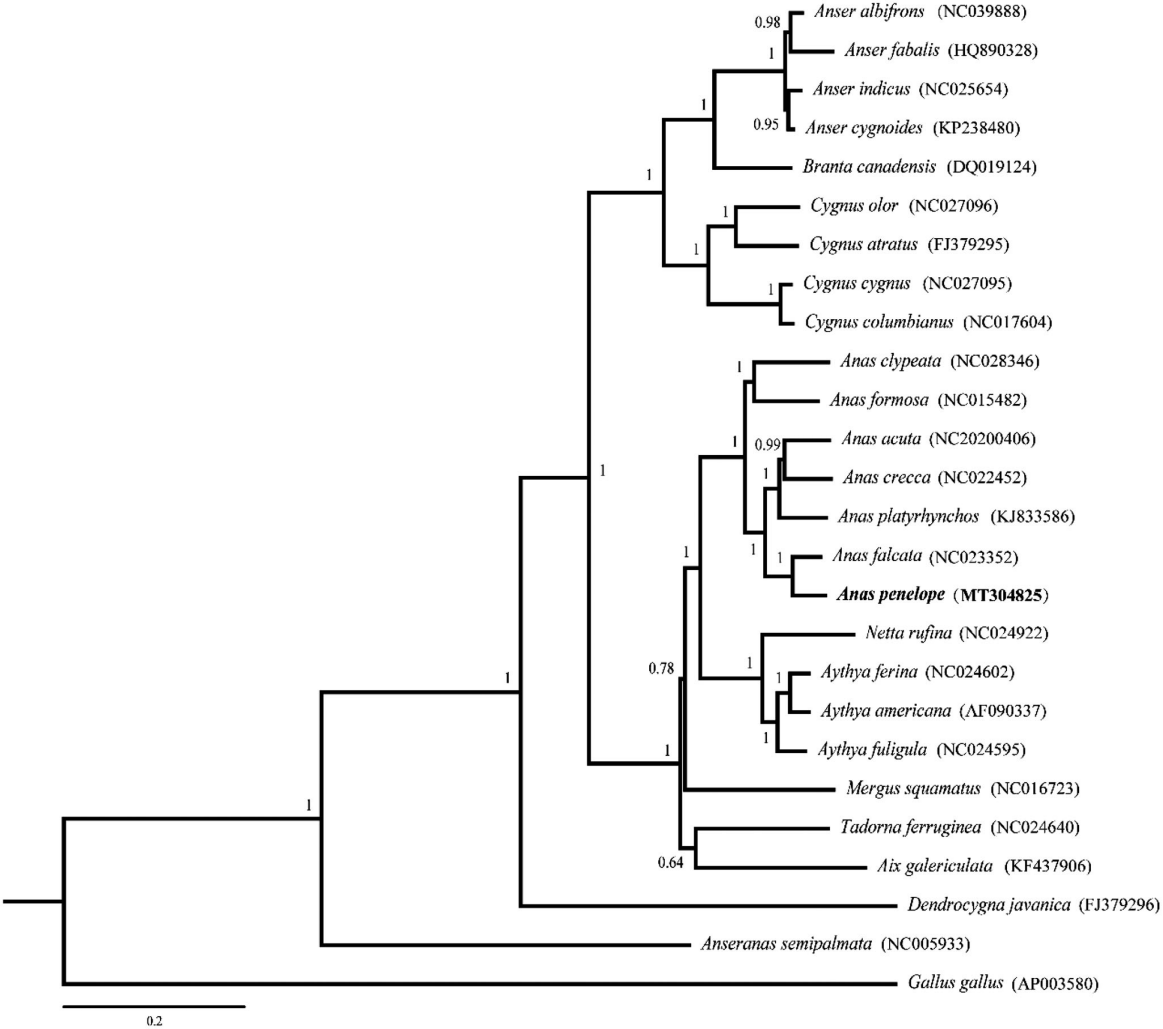
Phylogenetic relationship of *Anas peneope* and the other 25 species based on the Bayesian method. The bootstrap values are shown at the nodes.

## Data Availability

The data that support the findings of this study are openly availablein NCBI at www.ncbi.nlm.nih.gov, reference number (MT304825).
